# *Sedum lipingense* (Crassulaceae) identifying a new stonecrop species in SE Guizhou, China, based on morphological and molecular evidence

**DOI:** 10.3897/phytokeys.134.38287

**Published:** 2019-10-28

**Authors:** Ren-Bo Zhang, Tan Deng, Quan-Li Dou, Lin He, Xin-Yun Lv, Hong Jiang

**Affiliations:** 1 Department of Biology, Zunyi Normal College, Zunyi, Guizhou 563002, China Zunyi Normal College Zunyi China

**Keywords:** flora of Guizhou, karst, limestone flora, new taxon, *Sedum lipingense*

## Abstract

We describe and illustrate *Sedum lipingense* (Crassulaceae), a new species of stonecrop found in the limestone areas of SE Guizhou, China. Based on the presence of adaxially gibbous carpels and follicles, this taxon belongs to sect. Sedum S.H. Fu. The new species superficially resembles *S. subtile* Miquel and *S. bulbiferum* Makino but differs from these two taxa in its development of a basal leaf rosette during florescence. The nrDNA internal transcribed spacer (ITS) sequences also support the claim that this plant is a new species in the *Sedum* genus.

## Introduction

*Sedum* Linnaeus is the largest genus in the Crassulaceae family, containing about 430 species, with the greatest diversity centering in eastern Asia (Thiede and Eggli 2007, Ito et al. 2017a). Approximately 121 *Sedum* species (91 endemics) occur in China, and 49 of these species (34 endemics) belong to sect. Sedum, a subclass which possess adaxially gibbous carpels and follicles (Wu et al. 2013). There are 23 species within five genera of Crassulaceae found in the Guizhou Province (Li et al. 1985). From 2005, a number of new species of *Sedum* were reported across mainland China, in areas including Zhejiang (Wang et al. 2005, Jin et al. 2010, 2013), Anhui (Xie et al. 2014, Chen et al. 2017) and the Guizhou Province (Yang et al. 2012). In China, only a few species in this genus retain rosette leaves during florescence, such as *S. balfourii* Hamet and S. drymarioides var. saxifragiforme X. F. Jin & H. W. Zhang. *Sedum balfourii* was formerly placed in sect. Aizoon, within the genus *Sedum* (Fu and Fu 1984), but was then moved to the genus *Ohbaea* (Raymond-Hamet) V. V. Byalt & I. V. Sokolova (Fu and Ohba 2001) based on its conspicuous lateral flowering stems that derive from rosettes during florescence.

During our fieldwork, a new species of *Sedum* was discovered in Liping County, Qiandongnan Prefecture, Guizhou Province, China. This particular species has conspicuous rosettes during florescence, an attribute similar to *O. balfourii*. However, the new species differs from *O. balfourii* as it possesses central flowering stems rather than lateral ones (Fig. 2D). It also differs from S. drymarioides var. saxifragiforme, a species which is glandular-pubescent throughout, despite its rosette leaves. Based on its adaxially gibbous carpels, we place the new species in Sect. Sedum. Macro-morphological character studies indicated that this species is also somewhat similar to *S. subtile* Miquel and *S. bulbiferum* Makino, sharing a number of traits with these species, including opposite leaves on proximal stems and alternate leaves mainly on distal stems. We conducted morphological comparisons and molecular phylogenetic analysis to elucidate the presumed new *Sedum* species.

## Materials and methods

All morphological characters were measured using dissecting microscopes. Specimen checking was done at PE, IBK, ZY, with the additional use of some web database, including the Plant Photo Bank of China (http://ppbc.iplant.cn/) and Global Plants (http://plants.jstor.org/).

Leaf material from the presumed new species was collected in the field, and immediately dried in silica gel for DNA extraction. The nuclear ribosomal internal transcribed spacer (ITS) regions were used as molecular markers. ITS-F (TGAACCTGCGGAAGGATCAT) and ITS-R (GGTAGTCCCGCCTGACCTG) primers (Wu et al. 2013) were selected to amplify the ITS sequences. DNA extraction and PCR amplification of the new species followed the procedure of Wu et al. (2013). Primer synthesis and PCR product sequencing were carried out at the Shanghai Sangon Biotech Institute, China.

The ITS sequence of the new species, as well as the ITS sequences of the congeners downloaded from GeneBank (Table 1), were aligned using MEGA7 and then manually adjusted. Bayesian inference was implemented using MrBayes v3.2.6. Prior to the Bayesian analysis, the Akaike information criterion (AIC) implemented in mrModelTest v1.0 was used to select the best-fit model (GTR+I+G) of molecular evolution. For the BI analyses, four Markov Chain Monte Carlo (MCMC) chains were run, sampling one tree every 100 generations for 2,000,000 generations starting with a random tree (Xie et al. 2014). When the log-likelihood scores were found to have stabilized, a consensus tree was calculated after omitting 5,000 sampled trees as burn-in. *Aeonium lancerottense*, *A. viscatum* and *Greenovia aizoon* were selected as the outgroups referring to Ito et al. (2017b).

**Table 1.** Accession information relating to internal transcribed spacer (ITS) sequences downloaded from GeneBank.

Species	Voucher	Accession no.
*Aeonium lancerottense*	*MEM 1518*	AY082143
*Aeonium viscatum*	*MEM 1432*	AY082154
*Greenovia aizoon*	*MEM 1425*	AY082112
*Sedum alfredii*	*WUK415208*	FJ919953
*Sedum baileyi*	*LBG0064555*	FJ919935
*Sedum bergeri*	*Ni et al.*	AY352897
*Sedum bulbiferum_416*	*Ito416*	LC229234
*Sedum bulbiferum_hs41*	*130514hs41*	KM111166
*Sedum bulbiferum_qz09*	*130524qz09*	KM111165
*Sedum emarginatum*	*130512hs27*	KM111145
*Sedum erici-magnusii*	*Ito 2077*	LC229235
*Sedum erythrospermum*	*Tsutsumi 504*	AB906473
*Sedum formosanum*	*Ito 1260*	LC229279
*Sedum hakonense*	*S. Mayuzumi C00005*	AB088625
*Sedum hangzhouense*	*Ito2604 (TNS)*	LC260130
*Sedum japonicum*	*Kokubugata 16749*	AB906475
*Sedum jiulungshanense*	*CMQ20150076*	LC229243
*Sedum kiangnanense*	*Ito 1030*	LC229244
*Sedum lineare*	*Mayuzumi C00120*	AB088623
***Sedum lipingense***	*ZRB1479*	MN150061
*Sedum lungtsuanense*	*Ito3563*	LC260131
*Sedum makinoi*	*Kokubugata 16730*	AB906476
*Sedum mexicanum*	*Ito 647*	LC229247
*Sedum morrisonense*	*Ito2765*	LC229290
*Sedum multicaule*	*Miyamoto et al. TI9596136*	AB088631
*Sedum nagasakianum*	*Ito2064*	LC229249
*Sedum nokoense*	*Kokubugata 10426*	AB906478
*Sedum oligospermum*	*CMQ 74*	LC229250
*Sedum oreades*	*G. Y. Rao 090803-03*	KF113733
*Sedum polytrichoides*	*CMQ1057*	LC229251
*Sedum rupifragum*	*Ito 2070*	LC229254
*Sedum sarmentosum*	*Ito 978*	LC229255
*Sedum satumense*	*Ito2295*	LC229256
*Sedum trullipetalum*	*9420132*	AB088630
*Sedum subtile_1999*	*A. Shimizu 1999*	AB088622
*Sedum subtile_2259*	*Ito2259*	LC229257
*Sedum subtile_624*	*Ito 624*	AB930277
*Sedum taiwanianum*	*Ito2770*	LC229297
*Sedum tetractinum*	*Ito3623*	LC260135
*Sedum tianmushanense*	*LP 67*	LC229261
*Sedum tosaense*	*Kokubugata 16726*	AB906483
*Sedum triactina*	*9596091*	AB088629
*Sedum tricarpum*	*Ito 2269*	LC229259
*Sedum trullipetalum*	*Miyamoto et al.9420132*	AB088630
*Sedum truncastigmum*	*Ito3254*	LC229306
*Sedum yabeanum*	*S. Mayuzumi C00029*	AB088626
*Sedum zentaro-tashiroi*	*H. Ohba 1998*	AB088619

## Results

### Molecular analyses

In this study, the sequences of 40 species (44 samples) were treated as ingroups. Sequence length was 584 bp for the ITS region, of which 234 characters were constant, 45 characters were parsimony-uninformative and 305 characters were parsimony-informative.

**Figure 1.**
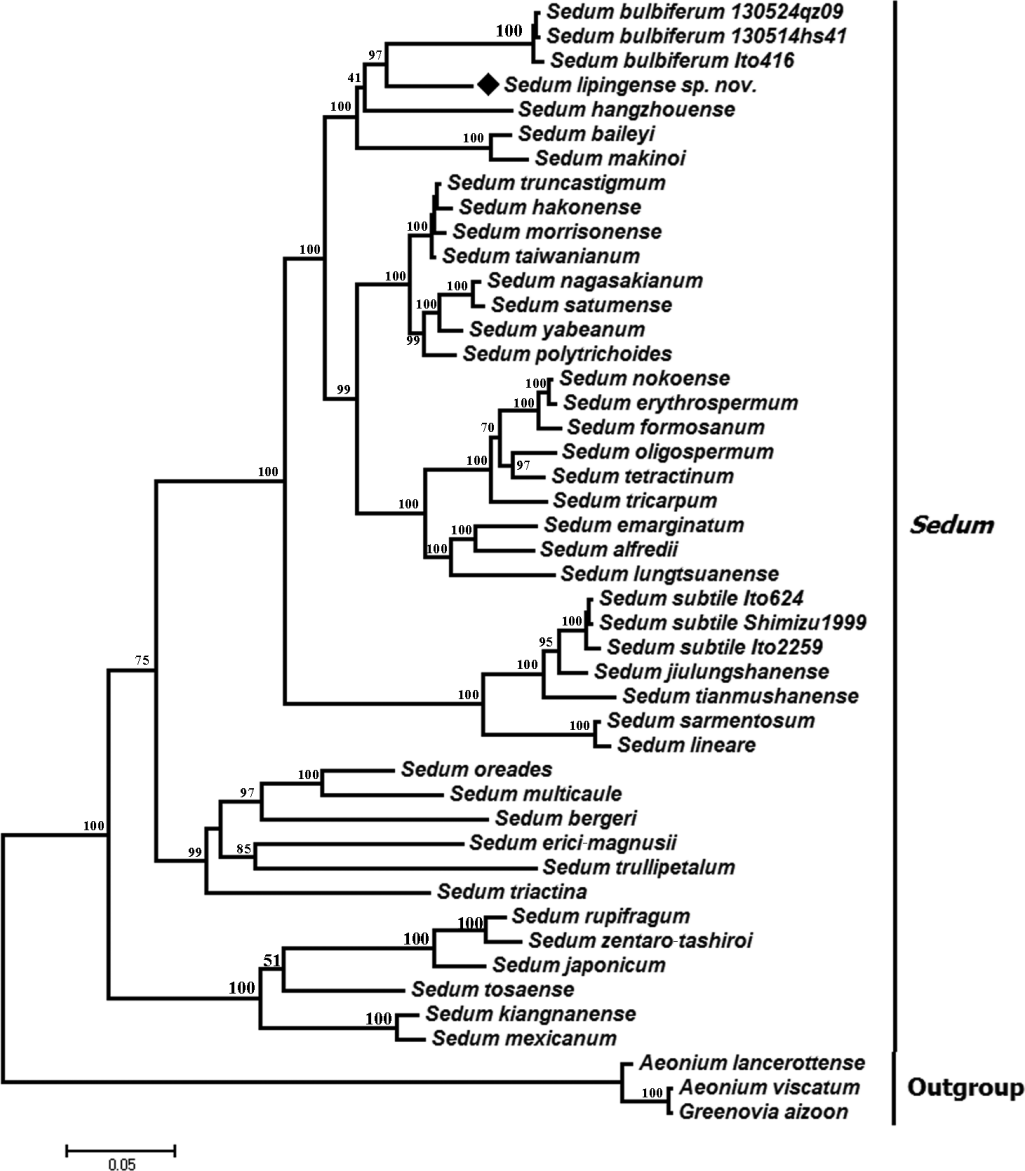
Bayesian phylogenetic tree based on ITS sequence for genus *Sedum* related to *S. lipingense* and three outgroups. Bayesian posterior probabilities are shown.

The sequence of the ITS region taken from *S. lipingense* aligned with the genus *Sedum*, confirming its generic identity (Fig. 1). The new species was resolved as sister to *S. bulbiferum* (Bayesian posterior probabilities (PP) was 97) but turned out to be genetically distant from *S. subtile.* There were 50 nucleotides differ between *S. lipingense* and *S. bulbiferum*, suggesting the high variation compared to the closest relatives was remarkable.

*S. lipingense* and *S. bulbiferum* were found to be nested with *S. hangzhouense* (PP = 41, suggesting a weak support), and then to be nested with *S. baileyi* and *S. makinoi* (PP = 100), all species with alternate or opposite stem leaves. Except for *S. lipingense*, the above four (or perhaps two-three) species were also clustered as a distinct clade (Wu et al. 2013, Xie et al. 2014, Ito et al. 2017a), suggesting that the four species are closely related. *Sedum lipingense* is a close member to this clade, but these species form a polytomy and it is hard to say for sure, which one is the closest relative of *S. lipingense.Sedum subtile* is not within the same clade as *S. bulbiferum*, *S. hangzhouense*, *S. baileyi*, and *S. makinoi* (Wu et al. 2013) or with *S. hangzhouense*, *S. baileyi*, and *S. makinoi* (Ito et al. 2017a), suggesting that the relationship between *S. subtile* and *S. lipingense* is relatively distant.

### Taxonomy

#### Sedum lipingense

Taxon classificationPlantaeSaxifragalesCrassulaceae

R.B. Zhang, D. Tan & R.X. Wei sp. nov.

8EE864DA-F7AE-5CA3-89A3-3134E83C145E

urn:lsid:ipni.org:names:77202732-1

Figs 2 , 3 , 4

##### Diagnosis.

*S. lipingense* can be distinguished from the closely related *S. subtile* and *S. bulbiferum* by the presence of rosettes, absent sterile shoots and bulbils, subequal lanceolate-oblong sepals, and other traits (Table 2).

**Table 2.** Comparing the diagnostics of *Sedum lipingense* sp. nov., *S. subtile* and *S. bulbiferum*.

Traits		*S. lipingense*	*S. subtile*	*S. bulbiferum*
Rosette leaves during florescence		**present**	absent	absent
Sterile shoots		absent	**present**	absent
Flowering stem		3–7 cm	5–10 cm	7–22 cm
Proximal stem leaves	Phyllotaxy	alternate, sometimes opposite on lateral flowering stem	opposite or 3–6-verticillate	opposite
	Leaf blade	broadly obovate	obovate	ovate-spatulate
Distal stem leaves	Phyllotaxy	alternate (sometimes subopposite)	alternate	alternate
	Leaf blade	spatulate-oblanceolate	oblanceolate-linear	spatulate-oblanceolate
	Bulbils in axils	absent	absent	**present**
Cymes	Branches	(2-) 3	2- or 3-branched	3-branched, branches 2-forked
	Branch flowers	**1- to two**	3- to several	many
Sepals		**lanceolate-oblong, subequal**	broadly linear to narrowly lanceolate, unequal	lanceolate to oblanceolate, unequal
Nectar scales		broadly cuneate, ca. 0.6 × 0.4 mm, apex truncate	broadly cuneate, ca. 0.4 × 0.5 mm, apex truncate	obovate, ca. 0.6 mm
Carpels		ca. 3.5 mm base connate for ca. 1 mm	ca. 5 mm base connate for ca. 2 mm	ca. 4 mm base connate for ca. 1 mm
Styles		ca. 1 mm	ca. 2 mm	ca. 1 mm
Fl.		Apr–May	Apr–Jun	Apr–May
Fr.		May–Jun	Jul–Aug	Jun–Jul

##### Type.

CHINA. Guizhou Province, Kaili City, Liping County, Mengyan Township, on moist rocks, 26°07'N, 108°42'E, 800 m alt., 13 April 2019, *ZRB1479* (fl., holotype ZY!, isotype IBK!), 16 June 2019, *ZRB1495* (fr., paratype ZY!)

##### Description.

Biennial (or perennial?) herb. ***Sterile stems*** absent. ***Rosette*** present during florescence; rosette leaves alternate, broadly obovate, base attenuated and shortly spurred, 0.5–1.5 × 0.4–0.7 cm. ***Flowering stems*** 1 to 3 (–4), erect, slender, 3–7 cm; single stems shoot from rosette centers, others shoot from the rosette leaf axils; lateral proximal leaves sometimes opposite, akin to rosette leaves but smaller, 0.6–0.8 × 0.3–0.5 cm, base shortly spurred; distal leaves alternate, spatulate-obovate to spatulate-oblanceolate, 0.7–1.2 × 0.3–0.4 cm, apex obtuse, base shortly spurred. ***Cymes*** scorpioid, 2 to 3 branched; branches 1 to 2 flowered; bracts obliquely oblanceolate, apex obtuse, 4–9 × 2–4 mm. ***Sepals*** 5, lanceolate-oblong, subequal, ca. 2 mm, base shortly spurred, apex obtuse. ***Petals*** 5, yellow, broadly lanceolate, ca. 4 mm, apex mucronate. ***Stamens*** 10; antesepalous one ca. 3 mm; antepetalous one inserted ca. 1 mm above petal base, slightly shorter than the antesepalous stamens. ***Nectar scales*** broadly cuneate, ca. 0.6 × 0.4 mm, apex truncate. ***Carpels*** erect, lanceolate, ca. 3.5 mm, base connate for ca. 1 mm. ***Styles*** slender, ca. 1 mm. ***Follicles*** stellately divergent at maturity. ***Seeds*** oblong, ca. 0.6 mm, papillate.

**Figure 2.**
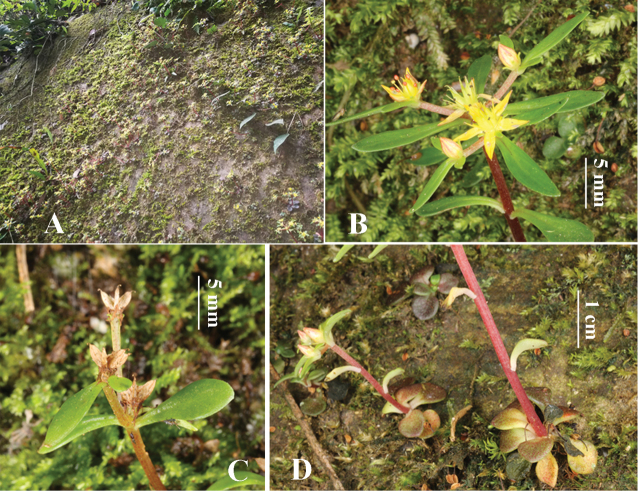
*Sedum lipingense***A** natural habitat **B** 3-branched scorpioid cyme **C** follicles and bracts **D** single flowering stems derived from rosette centers. Charted by Ren-Bo Zhang.

**Figure 3.**
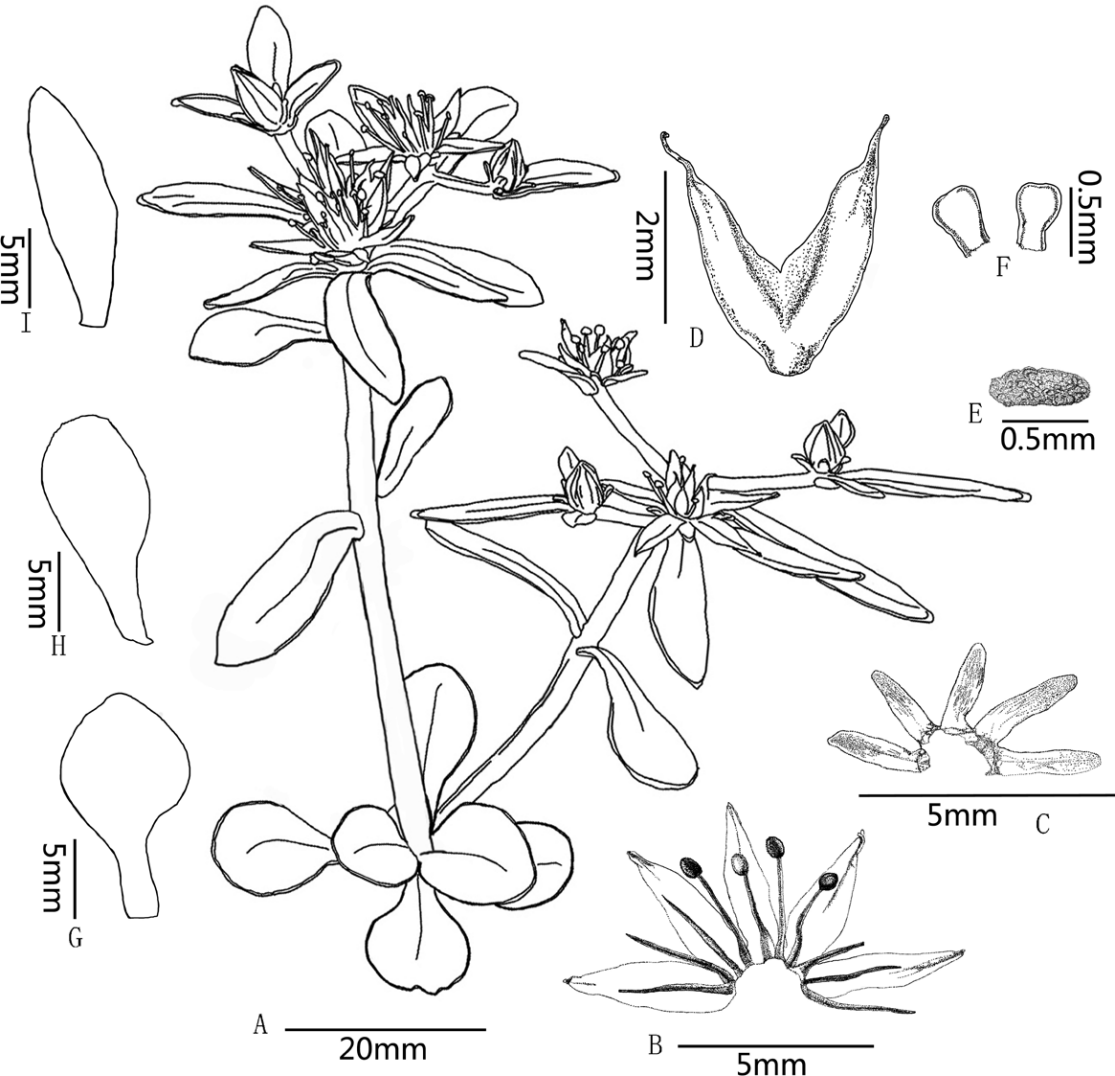
*Sedum lipingense***A** flowering plant **B** opened corolla **C** sepals **D** two follicles **E** seed **F** nectar scales **G** rosette leaf **H** distal leaf **I** bract of flower. Drawn by Tan Deng.

**Figure 4.**
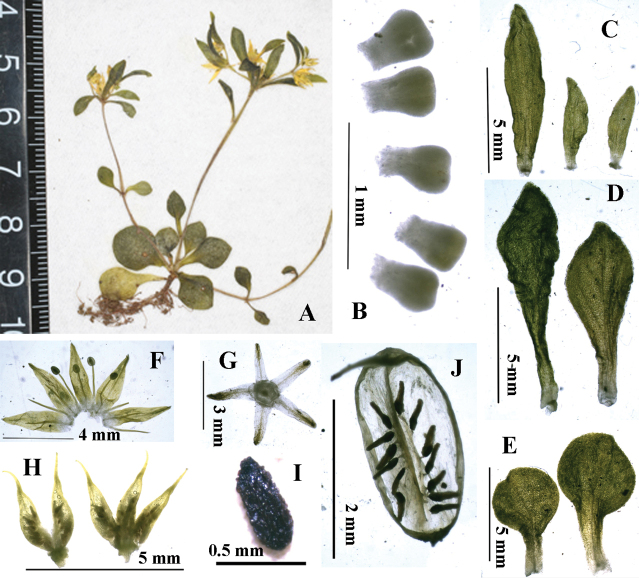
*Sedum lipingense***A** rosette, central and lateral flowering stems **B** nectar scales **C** bracts of flowers **D** distal leaves **E** rosette leaves **F** opened corolla **G** sepals **H** split carpels **I** seed. Charted by Ren-Bo Zhang.

##### Distribution and habitat.

At this time, based on our field observations, *Sedum lipingense* is only known to occur in Longxi village, Mengyan town, Liping County, Guizhou Province. It grows on moist limestone rocks, at ca. 800 m altitude, in groups of several hundred individuals.

##### Conservation status.

This species is currently known to occur in a single valley and we suggest its placement in the Data Deficient category of IUCN (2017).

##### Phenology.

This new species was observed flowering from April to May and fruiting from May to June.

##### Etymology.

The specific epithet ‘*lipingense*’ is derived from the plant’s locality, Liping County, Guizhou Province, China.

## Supplementary Material

XML Treatment for Sedum lipingense
